# Gerstmann-Sträussler-Scheinker disease subtypes efficiently transmit in bank voles as genuine prion diseases

**DOI:** 10.1038/srep20443

**Published:** 2016-02-04

**Authors:** Laura Pirisinu, Michele A. Di Bari, Claudia D’Agostino, Stefano Marcon, Geraldina Riccardi, Anna Poleggi, Mark L. Cohen, Brian S. Appleby, Pierluigi Gambetti, Bernardino Ghetti, Umberto Agrimi, Romolo Nonno

**Affiliations:** 1 Department of Veterinary Public Health and Food Safety, Istituto Superiore di Sanità, Viale Regina Elena 299, 00161, Rome, Italy; 2 Department of Cell Biology and Neurosciences, Istituto Superiore di Sanità, Viale Regina Elena 299, 00161, Rome, Italy; 3 Department of Pathology, National Prion Disease Pathology Surveillance Center, Case Western Reserve University, School of Medicine, 2085 Adelbert Road Cleveland, Ohio, OH 44106, USA; 4 Department of Pathology and Laboratory Medicine, Indiana University School of Medicine, Indianapolis, IN 46202, USA

## Abstract

Gerstmann-Sträussler-Scheinker disease (GSS) is an inherited neurodegenerative disorder associated with mutations in the prion protein gene and accumulation of misfolded PrP with protease-resistant fragments (PrP^res^) of 6–8 kDa. With the exception of a few GSS cases characterized by co-accumulation of PrP^res^ of 21 kDa, efforts to transmit GSS to rodents have been unsuccessful. As a result, GSS subtypes exclusively associated with 6–8 kDa PrP^res^ have often been considered as non-transmissible proteinopathies rather than true prion diseases. We show that GSS with P102L, A117V and F198S mutations transmit efficiently and produce distinct pathological phenotypes in bank voles *(M. glareolus*), irrespective of the presence of 21 kDa PrP^res^ in the inoculum, demonstrating that GSS is a genuine prion disease characterized by both transmissibility and strain variation.

Transmissible spongiform encephalopathies (TSEs), or prion diseases, are fatal neurodegenerative disorders associated with the accumulation of PrP^Sc^, a misfolded form of the cellular prion protein (PrP^C^). PrP^Sc^ is considered the main or exclusive component of infectious prions^1^. Creutzfeldt-Jakob disease (CJD) is the most common TSE in humans and may be inherited/genetic, acquired/iatrogenic, or sporadic. Inherited forms of human prion disease also include fatal familial insomnia (FFI) and Gerstmann–Sträussler–Scheinker disease (GSS), caused by pathogenic mutations in the prion protein gene *(PRNP)* believed to predispose mutant PrP^C^ to convert spontaneously to PrP^Sc^.

GSS is an autosomal dominant prion disease associated with point mutations in the prion protein gene (*PRNP*) and characterized pathologically by the deposition of prion protein amyloid in the brain accompanied by gliosis, neuronal loss, and variable amounts of spongiform degeneration^2^.

As a genetically encoded prion disease, GSS could provide the strongest evidence that the abnormal prion protein is the causative agent of prion disease. However, lack of successful transmission of several forms of GSS has cast significant doubt on this hypothesis^3^,^4^,^5^,^6^.

In most TSEs, proteinase K (PK) treatment of PrP^Sc^ results in PK-resistant aggregates (PrP^res^) composed of variably glycosylated C-terminal PrP^res^ fragments, whose non-glycosylated PrP^res^ show molecular weights of 19-to-21 kDa^7^. In contrast, GSS subtypes are characterized by non-glycosylated PrP^res^ composed of PrP internal fragments, truncated at N- and C- termini, whose molecular weight varies between 6 and 8 kDa, depending on the specific *PRNP* mutation^8^,^9^,^10^. The most frequent GSS mutation is a proline-to-leucine substitution at *PRNP* codon 102 (P102L). The GSS P102L phenotype was reported to be highly heterogeneous, and seminal studies suggested a correlation between pathological and molecular variability in these cases^8^. Indeed, two distinct phenotypes of GSS P102L have been described, with the accumulation of both 21 and 8 kDa PrP^res^ in patients with spongiform degeneration, and 8 kDa PrP^res^ only in patients lacking spongiform degeneration^8^,^11^. Importantly, transmission studies suggested that the 21 kDa PrP^res^ isoform was responsible for the biological properties of GSS, as only cases with 21 kDa PrP^res^ were transmissible in wild-type mice^6^ and transgenic mice (Tg101LL) expressing mutated murine PrP equivalent to human P102L^12^, and always induced 21 kDa PrP^res^ in the recipient hosts. Brain extracts from GSS P102L patients with 8 kDa PrP^res^ were not transmissible to wild-type mice, and induced PrP-amyloid deposition but did not replicate the TSE infectious agent in Tg101LL mice^5^. Similarly, GSS cases with *PRNP* mutations exclusively associated with atypical 6–8 kDa internal PrP^res^ fragments have not been convincingly shown to induce prion propagation in the brains of new hosts after experimental challenge. Among these, GSS cases associated with A117V and F198S mutations show atypical PrP^res^ fragments of 6–7 kDa and 8 kDa, respectively^8^,^9^,^10^,^13^. Several patients with the latter mutation variant were identified in a large Indiana kindred^14^ and are characterized by prominent PrP plaque deposition and tau-positive fibrillary tangles^15^,^16^,^17^. GSS A117V cases show a wide range of clinical and pathological presentations even within the same family^18^,^19^,^20^,^21^. A recent study reported partial transmission of GSS A117V cases to transgenic mice over-expressing the human 117V PrP, characterized by low clinical attack rates, long incubation periods (>600 days), and accumulation of inherently unstable PrP^Sc^ aggregates^22^.

Inefficient transmission of GSS cases with 6–8 kDa PrP^res^ has led to the hypothesis that GSS is a non-transmissible proteinopathy rather than authentic prion disease^5^, implying that proteinopathies and prion diseases were associated with different isoforms of PrP^Sc^. Indeed, recent studies suggest differences between cell-to-cell spread of misfolded proteins and the transfer of infectivity from one organism to another^23^, as well as between pathogenic and infectious properties of the prion protein itself 24. To address these issues, we attempted to transmit several biochemically characterized GSS cases^10^ to bank voles, a rodent model able to propagate most human and animal prion diseases^25^,^26^,^27^,^28^,^29^,^30^,^31^,^32^,^33^. We found that all GSS cases induced prion diseases characterized by spongiform degeneration, PrP^Sc^ deposition, and propagation of infectious agents in the brain of recipient voles, implying that GSS, similar to others TSEs, is associated with infectious prions.

## Results

### GSS with A117V mutation

**Figure 1:**
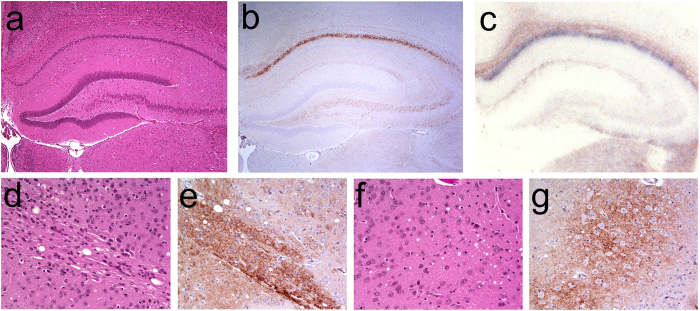
Histological and immunohistochemical analysis of Bv109I infected with A117V. Sections of the hippocampus (**a**–**c**), the anterior commissure (**d**,**e**) and the piriform cortex (**f**,**g**) were analyzed by haematoxylin & eosin staining (**a**,**d**,**f**), by immunohistochemistry for PrP^Sc^ (**b**,**e**,**g**) and by PET-blot for protease-resistant PrP^Sc^ (**c**).

**Figure 2:**
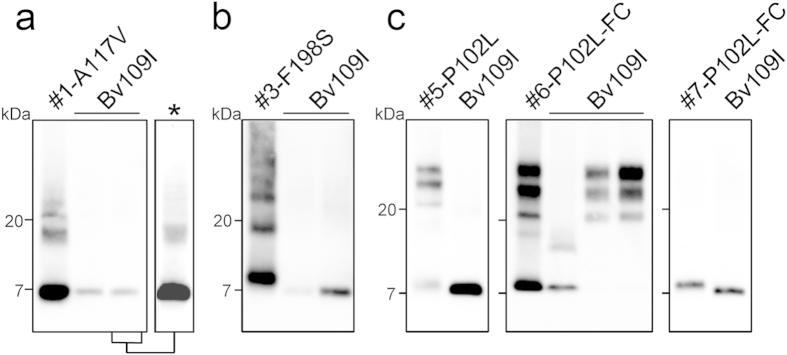
Representative Western blot of PrP^res^ from Bv109I inoculated with GSS cases. (**a**) PrP^res^ from Bv109I inoculated with an A117V case in comparison with the starting inoculum; for a lane with PrP^res^ from Bv109I a longer exposure of the blot is also included (asterisk). (**b**) PrP^res^ from Bv109I inoculated with a F198S case in comparison with the starting inoculum. (**c**) PrP^res^ from Bv109I inoculated with P102L cases #5 (left panel), #6 (middle panel) and #7 (right panel), along with the starting inocula. Note that Bv109I inoculated with case #6 accumulated either small PrP^res^ fragments (lane 2, middle panel) or 21 kDa PrP^res^ (lanes 3 and 4). All membranes were probed with 9A2.

Brain homogenates from two unrelated GSS cases with A117V mutation (Supplementary Table S1), both associated with small internal PrP^res^ fragments of 6–7 kDa^10^, were inoculated in bank voles carrying isoleucine at *PRNP* codon 109 (Bv109I). Remarkably, both cases caused a rapidly progressive and fatal neurological disease, with mean survival time of ~95 days post-inoculation (dpi) (Table 1). In all clinically-affected voles, neuropathological analysis showed spongiform degeneration, gliosis, and neuronal loss (Fig. 1). In the white matter (Fig. 1a,d), spongiform changes mainly involved the alveus, dorsal hippocampal commissure, anterior commissure, white matter bundles in striatum and pallidum and, to a lesser extent, the corpus callosum. Spongiform changes in grey matter appeared as microvacuoles in hippocampus and cerebral cortex (Fig. 1a,f), particularly in motor, piriform and entorhinal cortices. Variable involvement of cerebellar molecular layer was also observed. By immunohistochemistry, PrP^Sc^ deposition was mainly found in areas with spongiform changes, particularly in the alveus and dorsal hippocampal commissure (Fig. 1b). PrP^Sc^ deposits appeared as coarse, granular deposits in white matter tracts (Fig. 1e) and synaptic/punctuate deposits in grey matter areas (Fig. 1g) and were resistant to PK (Fig. 1c). Brain homogenates from diseased voles contained protease-resistant PrP^Sc^ easily detectable in standard western blot as small internal PrP^res^ fragments of ~7 kDa truncated at both N- and C- termini (Supplementary Fig. S1). Notably, the PrP^res^ electrophoretic pattern in voles inoculated with GSS A117V cases was very similar to that in the human inocula (Fig. 2a).

Intriguingly, sub-passage in voles showed survival times (Table 1) and neuropathological patterns (Supplementary Fig. S2) similar to those observed after primary transmission, suggesting that the human-to-vole species barrier did not affect the efficiency of transmission of GSS A117V.

### GSS with F198S mutation

**Figure 3:**
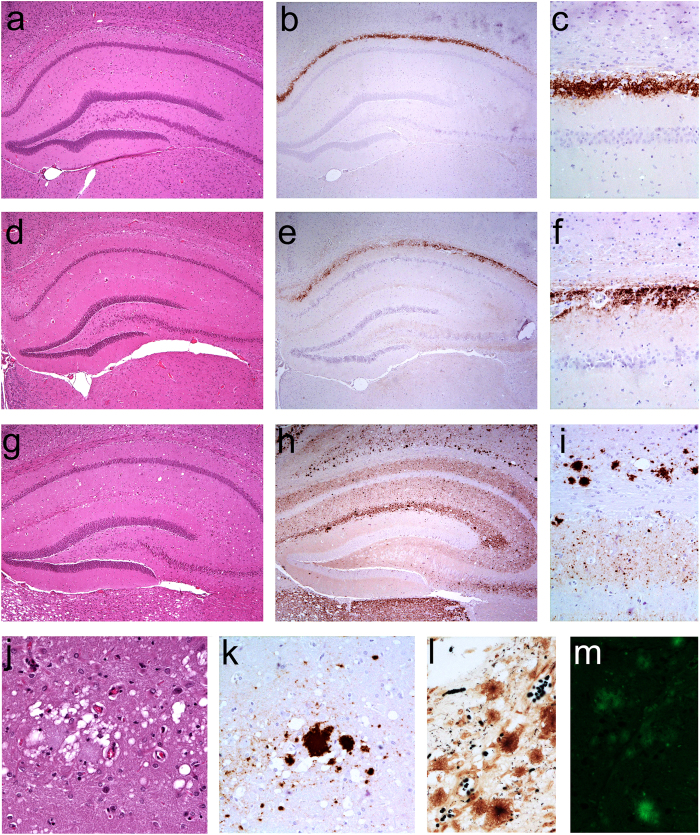
Histological and immunohistochemical analysis of Bv109I infected with F198S and P102L. Serial sections of hippocampus from Bv109I inoculated with F198S (**a**,**b**) and P102L (**d**,**e**,**g**,**h**) were analyzed by haematoxylin and eosin staining (**a**,**d**,**g**) or by immunohistochemistry for PrP^Sc^ (**b**,**e**,**h**). Panels (**c**,**f**,**i**) show higher magnifications from sections in panels (**b**,**e**,**h**). Sections in panels (**a**-**f**) show the hippocampus from Bv109I that accumulated 7–8 kDa PrP^res^, those in panels (**g**–**i**) show the hippocampus from Bv109I that accumulated 21 kDa PrP^res^. Panels (**j**,**k**,**l**,**m**) show higher magnifications of florid plaques in the stratum lacunoso-moleculare of the hippocampus (**j**) and cerebral cortex (**k**,**l**,**m**) from Bv109I that accumulated 21 kDa PrP^res^, stained with haematoxylin and eosin (**j**), immunohistochemistry for PrP^Sc^ (**k**), Bielschowsky silver staining (**l**) and Thioflavin-S (**m**).

Brain homogenates from two F198S patients from the same kindred (Supplementary Table S1) were inoculated into Bv109I. Case #3 caused fatal neurological disease in 14/15 animals after a mean survival period of 143 dpi (Table 1). In contrast, case #4 transmitted the disease in only one vole that showed terminal neurological disease after 153 dpi (Table 1). All other inoculated Bv109I were still alive and healthy after 800 dpi, or were sacrificed for intercurrent disease and found negative for PrP^Sc^. The lower amount of PrP^res^ detected in the inoculum from case #4 compared to case #3 might explain the different attack rates (Supplementary Fig. S3). In all affected voles, neuropathological, immunohistochemical and western blot analyses revealed PrP^res^ molecular signature (Fig. 2b), spongiform degeneration (Fig. 3a) and PrP^Sc^ deposition patterns (Fig. 3b,c) similar to those observed in Bv109I inoculated with GSS A117V. Direct comparison of PrP^res^ types revealed a downward shift from ∼8 to ~7 kDa in vole-passaged PrP^Sc^ compared to the human inocula (Fig. 2b).

### GSS with P102L mutation

To investigate whether distinct PrP^res^ patterns observed in GSS P102L cases correlated with biological properties other than pathological phenotypes, we selected brain tissues from three unrelated GSS P102L cases, either methionine homozygous or heterozygous at *PRNP* codon 129: case #5, with both 21 kDa and 8 kDa PrP^res^; case #6, with 21 kDa and 8 kDa PrP^res^ in cerebral cortex and only 8 kDa PrP^res^ in cerebellum; and case #7, with only 8 kDa PrP^res^ in all affected areas (Supplementary Table S1 and Supplementary Fig. S3).

Brain homogenate from frontal cortex of case #5 caused terminal disease in 6/8 animals after mean survival time of 363 dpi (Table 1). Despite that the original inoculum contained both 21 and 8 kDa PrP^res^ types, only ∼8 kDa PrP^res^ was found in affected voles (Fig. 2c).

Of case #6, we selected brain homogenates from frontal cortex, with 21 and 8 kDa PrP^res^, and from cerebellum, with only 8 kDa PrP^res^ (Supplementary Fig. S3). The cerebellar inoculum resulted in partial attack rate and short survival times, with Bv109I showing terminal neurological disease at 153 ± 9 dpi (Table 1) and accumulation of ~7–8 kDa PrP^res^ only. In contrast, the frontal cortex inoculum from the same case induced two distinct phenotypes: one Bv109I was affected at 265 dpi and showed only ~7–8 kDa PrP^res^, while seven voles showed clinical signs much later (at ~700 dpi) and accumulated 21 kDa PrP^res^ (Fig. 2c).

Finally, both frontal cortex and cerebellum from case #7 (characterized by the presence of only 8 kDa PrP^res^) transmitted very efficiently, causing disease in 14/16 animals at 208 ± 58 dpi and in 13/13 voles at 171 ± 53 dpi, respectively (Table 1). By western blot, all vole brains showed PrP^res^ fragments of ~7–8 kDa (Fig. 2c).

The neuropathological pattern in affected voles accumulating 7–8 kDa PrP^res^ was similar in all cases and was characterized by spongiform degeneration with prominent involvement of white matter tracts, hippocampus and cerebral cortices, similar to voles inoculated with A117V and F198S GSS (Fig. 3d–f). Voles challenged with case #6 P102L that accumulated 21 kDa PrP^res^ displayed a remarkably different neuropathological and immunohistochemical pattern. Medium and large vacuoles were found in several areas of grey matter (Fig. 3g,j), mainly involving thalamus, striatum, hippocampus, geniculate nucleus, and cerebral cortex, with much less white matter involvement. By immunohistochemistry, punctuate PrP^Sc^ deposits were mainly found in cerebral cortex, hippocampus (Fig. 3h,i), thalamus and striatum. In some animals, we also observed florid plaques in thalamus, hippocampus (Fig. 3j) and cerebral cortex (Fig. 3k–m). Immunohistochemistry for PrP^Sc^ (Fig. 3k), Bielschowsky silver staining (Fig. 3l) and Thioflavin-S fluorescence (Fig. 3m) confirmed that these were indeed PrP-amyloid plaques. Furthermore, PET-blot analysis showed that plaques consisted of protease resistant PrP^Sc^ (Supplementary Fig. S4).

### Biochemical phenotypes of PrP^Sc^ in GSS-inoculated voles

**Figure 4:**
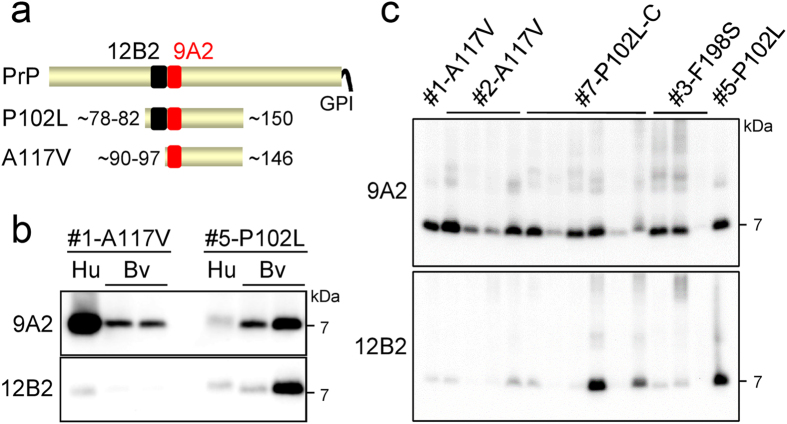
Different internal PrP^res^ fragments in voles inoculated with GSS cases. (**a**) schematic representation of PrP^c^ and PrP^res^ fragments with epitopes of mAbs 12B2 and 9A2 that allow discrimination of N-terminal PK cleavage sites of P102L and A117V PrP^res^ fragments. (**b**) Western blot of PrP^res^ fragments from Bv109I (Bv) inoculated with A117V (case #1) and P102L (case #5), along with the respective human inocula (Hu). Replica blots were probed with 9A2 and 12B2. (**c**) Western blot of PrP^res^ fragments from Bv109I inoculated with GSS cases. Replica blots were probed with 9A2 and 12B2 to discriminate PrP^res^ types based on N-terminal PK cleavage. Bv109I samples analyzed in panel (**b**) were used as internal controls in panel (**c**), with A117V-infected Bv in the first lane and P102L-infected Bv in the last lane. In this representative set of samples, the 12B2 epitope was mainly lost in voles infected with A117V and F198S and in all but two voles infected with P102L case #7.

We noticed that in some experiment the ~7–8 kDa PrP^res^ observed in voles infected with P102L GSS appeared slightly larger than PrP^res^ from A117V-infected voles by direct comparison in western blot (data not shown). We recently reported that PrP^res^ types associated with P102L and A117V mutations can be discriminated by the presence of the N-terminal epitope of mAb 12B2 (amino acid 83–89, human numbering), which is mostly cleaved by PK in A117V cases, but not in P102L (Fig. 4a,b)^10^, so we directly compared PrP^res^ from human inocula (cases #1 and #5) and recipient voles in replica blots probed with mAbs 9A2 and 12B2, directed to flanking epitopes in the N-terminus of PrP^res^ (Fig. 4a). We found that PrP^res^ from voles matched their human counterparts, showing faithful propagation of the PrP^res^ N-terminal PK-cleavage after passage in voles (Fig. 4b), suggesting that vole-passaged GSS A117V or P102L cases might have conformationally distinct PrP^Sc^ distinguishable by the presence or absence of the 12B2 epitope in PrP^res^, resulting in ~7 kDa and ~8 kDa PrP^res^ fragments in vole-passaged GSS cases #1 and #5, respectively.

**Figure 5:**
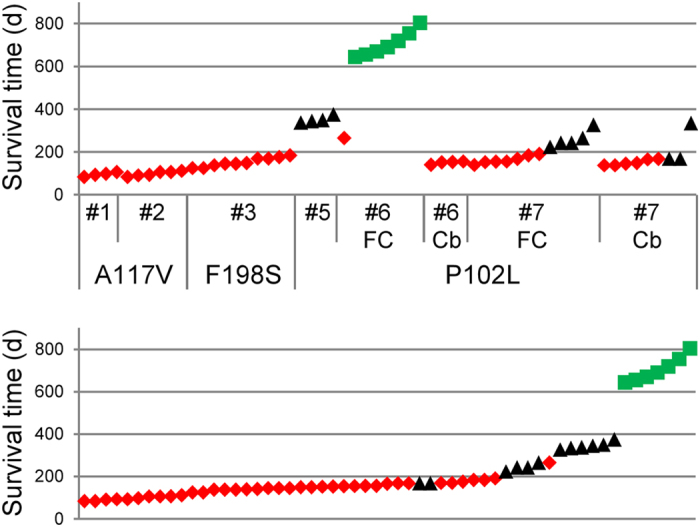
Summary of the different PrP^res^ types detected in voles inoculated with GSS cases. The upper graph shows the PrP^res^ types (symbols) and survival times (*y* axis) of Bv109I inoculated with GSS cases as indicated (*x* axis). Red symbols indicate 12B2-negative PrP^res^ of ~7 kDa, black symbols indicate 12B2-positive PrP^res^ of ~8 kDa, and green symbols indicate 21 kDa PrP^res^. The same data and symbols were used for the graph in the lower panel, which shows the PrP^res^ type of individual Bv109I ordered by their survival time irrespective of the group inocula. The survival time varied significantly depending on their PrP^res^ type (ANOVA, P < 0.0001). By two-tailed *t*-test comparisons, voles with ~7 kDa PrP^res^ showed a survival time shorter than those with ~8 kDa PrP^res^ (P < 0.0001) or with 21 kDa PrP^res^ (P < 0.0001), and voles with ~8 kDa PrP^res^ showed a survival time shorter than those with ~21 kDa PrP^res^ (P < 0.0001).

We used the 12B2 antibody to further characterize the internal PrP^res^ fragments accumulated in most of the GSS-infected bank voles. PrP^res^ from all voles inoculated with A117V and F198S showed the same molecular weight (~7 kDa) and the same N-terminal cleavage site (depicted by the partial loss of 12B2 mAb epitope in Fig. 4c). In contrast, voles inoculated with P102L cases contained PrP^res^ fragments with the 12B2 epitope, with some brains also showing PrP^res^ fragments without the 12B2 epitope, similar to those observed in brains of A117V- and F198S-inoculated voles (Fig. 4c). Figure 5 summarizes our biochemical typing of PrP^res^ in GSS-infected bank voles. Overall, the PrP^res^ type did not vary among voles infected with A117V or F198S GSS cases, while we observed three different molecular types of PrP^Sc^ in voles infected with P102L, characterized by PrP^res^ of 7 kDa, 8 kDa or 21 kDa PrP^res^ (Fig. 5, upper panel). Different PrP^res^ types were also identified among voles inoculated with the identical inocula: case #6 produced either 7 kDa or 21kDa PrP^res^; case #7 produced either 7 or 8 kDa PrP^res^ (Fig. 5, upper panel). Amalgamating all of our data, we found a correlation between survival periods and PrP^Sc^ types (Fig. 5, lower panel): 7 kDa PrP^res^ accumulated in bank voles with the short survivals, 8 kDa PrP^res^ was observed in voles with intermediate survivals, and 21 kDa PrP^res^ was observed only in very long survivors. This correlation suggests that each of the three PrP^res^ molecular types encipher distinct biological properties.

## Discussion

GSS has been at the center of a long debate among pathologists and prionologists based on atypical molecular and pathological features, broad variability of disease phenotypes (sometimes even within the same family)^20^,^21^,^34^,^35^,^36^ and inconsistent transmissibility in experimental models. Indeed, notwithstanding a recent report of low efficiency transmission of GSS A117V in transgenic mice expressing homologous human 117V PrP^22^, there is no definitive evidence that GSS phenotypes with 8 kDa PrP^res^ can transmit disease and propagate infectious prions.

We inoculated seven distinct GSS cases into Bv109I and observed disease transmission in all cases. Overall, 85/113 voles challenged with brain homogenates from different GSS patients developed a fatal neurological illness, most of them between 3 and 7 months post-challenge. Infected voles showed all cardinal neuropathological and molecular features of prion diseases, including spongiform degeneration and deposition of PK-resistant PrP^Sc^. Furthermore, GSS A117V infected vole brains were able to induce the same disease phenotype in recipient voles within 3–4 months after challenge, proving that a prion agent propagated in the brains of infected animals. These findings imply that brains of GSS patients harbor infectious prions with transmissibility features similar to those found in other human and animal TSEs.

Why GSS replication in recipient hosts has remained elusive is not clear. The ability to propagate heterologous PrP species seems to be especially limited for GSS associated with 8 kDa PrP^res^. Indeed, beside the reported inability of GSS with 8 kDa PrP^res^ to infect primates, mice and transgenic mouse lines, only mutant proteins were found in 8 kDa PrP^res^ fragments from patients with P102L mutation^11^,^37^ suggesting inefficient templating of 8 kDa PrP^res^ even with wild type human PrP. In this context, bank vole PrP appears to have been pivotal for proving the transmissibility of GSS. Accumulating evidence suggests that the remarkable susceptibility of bank voles to TSEs is primarily due to peculiar features of the bank vole PrP amino acid sequence. Transgenic expression of bank vole PrP in mice conferred a vole-like susceptibility to several TSEs^38^, and transgenic mouse lines over-expressing bank vole PrP with isoleucine at codon 109 spontaneously develop a transmissible neurological disease^39^. Some studies suggest that the presence of asparagines in or near the β2-α2 loop might create a permissive PrP sequence able to overcome some species barriers^25^,^30^,^40^. The unexpected lack of transmission barrier for human GSS A117V, despite several amino acid mismatches between human and vole PrPs, supports this hypothesis.

P102L, A117V and F198S GSS cases displayed varying transmission efficiencies. Brain extracts from two A117V mutation patients induced a highly aggressive TSE in voles, which faithfully reproduced the PrP^Sc^ molecular feature of the human counterpart and propagated without transmission barrier, providing definitive evidence for transmissibility of this GSS subtype. In the recent study reporting partial transmission of GSS A117V, the authors explained the low efficiency of transmission as resulting from instability of A117V-derived abnormal PrP, which was found to be labile and detectable in Tg mouse brains only using suitable conditions^22^. In contrast, we found PrP^Sc^ accumulated in brains of voles easily detectable in western blots after standard PK digestion, even after repeated freeze-thawing (data not shown), similar to that found in brain tissues from A117V patients (this study and ref. 10). These differences might be due to the specific GSS cases investigated, or to variation in western blot conditions between the two studies. However, it is also noteworthy that Asante and colleagues^22^ used a mouse line over-expressing PrP, providing a stoichiometric context for PrP^Sc^ aggregation and propagation different from that in human and vole brains, which may have affected the stability and propagation characteristics of PrP^Sc^ conformers.

We also present here the first evidence that F198S GSS cases produce transmissible prions, although the ability of our two cases to induce disease in bank voles was clearly different (Table 1). This finding was unexpected, especially in light of their overlapping biochemical features^10^, although the lower amount of PrP^res^ in case #4 compared to case #3 could provide a potential explanation for the observed discrepancy. Another explanation might be that case #3 was valine homozygous at codon 129, a molecular feature associated with early onset of disease^14^, while case #4 was methionine/valine heterozygous.

Based on previous evidence that GSS P102L cases with 21 kDa PrP^res^, but not with 8 kDa PrP^res^ only, were associated with “true” infectivity^5^, we analyzed three P102L cases representing the spectrum of molecular variability observed in P102L patients. Our results indisputably show that all cases were transmissible in voles. Close scrutiny of the transmission efficiencies of the various inocula ruled out any major role of 21 kDa PrP^res^ in determining transmissibility. In fact, shorter survival times and higher transmission rates were observed in voles inoculated with tissues containing 8 kDa PrP^res^ only (cerebellum of case #5 and brain tissues from case #6) compared with those inoculated with tissues containing both, 21 kDa and 8 kDa PrP^res^ (Table 1), indicating that PrP^Sc^ species generating 8 kDa PrP^res^ fragments are also associated with prion infectivity in P102L GSS. Interestingly, an inoculum containing both 21 kDa and 8 kDa PrP^res^ types (frontal cortex from case #6), resulted in the propagation of 7 kDa PrP^res^ in an individual vole after a short survival period, and of 21 kDa PrP^res^ in the remaining voles after much longer survival times, suggesting that PrP^Sc^ types associated with 21 kDa or 7–8 kDa PrP^res^ might behave as independent transmissible PrP^Sc^ species.

Even taking into consideration the variable presence of 21 kDa and 8 kDa PrP^res^ species in the GSS P102L patients studied, it is difficult to explain the variable efficiency of transmission that we observed. Tissues that induced the highest transmission rates were not those with the highest PrP^res^ content (compare data in Supplementary Fig. S3 and Table 1). All of our P102L cases segregated with methionine at codon 129 on the mutant allele but carried either valine (#5 and #7) or methionine (#6) at codon 129 of the unmutated PrP allele, which may have contributed to transmission variability. Indeed, it has been shown that PrP^Sc^ deriving from the non mutated allele contributes to molecular variability observed in P102L cases and increase the spectrum of PrP^Sc^ conformers^37^,^41^. Within the limited set of P102L GSS patients studied here at least three different molecular phenotypes were derived in voles, which partially matched those present in the respective inocula (Fig. 4c). Although subpassages are required to establish if different strains evolved in voles, the distinct neuropathological pattern observed in voles with 21 kDa PrP^res^ and the correlation between PrP^res^ type and survival time (Fig. 4d) support the hypothesis that GSS patients might harbor different prion strains, which could contribute to clinical and pathological heterogeneity. As Bv109I appears to be the first animal model able to faithfully propagate GSS, further investigation of the relationships between clinicopathological phenotypes, PrP^Sc^ conformers, and prion strains in families with GSS is warranted.

## Methods

### Human inocula

Patients were referred to the National Prion Disease Pathology Surveillance Center, Cleveland, OH (ID #1–4, 6, 7) and to the Italian National Registry of CJD at the Istituto Superiore di Sanità, Rome, Italy (ID #5). Clinicopathologic features, PrP polymorphisms and mutations, and brain areas sampled for analysis are reported in Supplementary Table S1. Coronal sections of human brain tissues were obtained at autopsy and stored at −80 °C until use. Informed consent to use human autopsy material for research purposes was obtained from all subjects. Clinical data and relevant hospital records were coded and handled according to the protocols approved by the Ethical Committee and Institutional Review Board of Case Western Reserve University to protect patients’ identities. The use human autopsy material for research has been approved by UHCMC IRB #05-14-09. Data collection and use of material for GSS case #5 was approved by the Ethical Committee of the Istituto Superiore di Sanità.

### Animals

Bv109I voles were obtained from the breeding colony at the Istituto Superiore di Sanità. The research protocol, approved by the Service for Biotechnology and Animal Welfare of the ISS and authorized by the Italian Ministry of Health, adhered to the guidelines contained in the Italian Legislative Decree 116/92, which transposed the European Directive 86/609/EEC on Laboratory Animal Protection, and then in the Legislative Decree 26/2014, which transposed the European Directive 2010/63/UE on Laboratory Animal Protection.

### Preparation of brain homogenates and bioassays

Tissues were homogenized at 10% (w/v) in phosphate buffered saline (PBS) and stored at -80 °C. Groups of eight-week-old Bv109I were inoculated intracerebrally with 20 μl of homogenate into the left cerebral hemisphere, under ketamine anaesthesia (ketamine 0.1 μg/g). All animals were individually identified by a passive integrated transponder. The animals were examined twice a week until neurological signs appeared, after which they were examined daily. Diseased animals were culled with carbon dioxide at the terminal stage of the disease, but before neurological impairment was such as to compromise their welfare, in particular their ability to drink and feed adequately. Survival time was calculated as the interval between inoculation and culling or death. ANOVA and unpaired t test statistical analyses for survival times of voles were carried out by GraphPad Prism software.

The brain from each animal was removed and cut sagittally into two parts: one stored at −80 °C and one fixed in formalin.

### Neuropathology

Histology, immunohistochemistry and PET-blot analysis were performed on formalin-fixed tissues as previously described^27^,^29^. Briefly, brains were trimmed at standard coronal levels, embedded in paraffin wax, cut at 6 μm and stained with haematoxylin and eosin, 0.05% Thioflavin-S, or Bielschowsky silver. PrP immunolabeling in immunohistochemistry and PET-blot was performed using the 6C2 mAb (epitope on bank vole PrP 111–116). Glial fibrillary acidic protein (GFAP) immunolabelling was performed with polyclonal rabbit anti-cow GFAP (1:500, Dako-Cytomation).

### PrP^res^ analysis and western blotting

Brain homogenates (20% w/v) were prepared as previously described^31^. After adding an equal volume of 100 mM Tris-HCl containing 4% sarkosyl, the homogenates were incubated for 30 min at 37 °C with gentle shaking. Proteinase K (Sigma-Aldrich) was added at a final concentration of 100 μg/ml and then the samples were incubated for 1 h at 55 °C with gentle shaking. Protease treatment was stopped with 3 mM PMSF (Sigma-Aldrich). Aliquots of samples were added with an equal volume of isopropanol/butanol (1:1 v/v) and centrifuged at 20,000 g for 5 min. Supernatants were discarded and the pellets were resuspended in denaturing sample buffer (NuPAGE LDS Sample Buffer, Invitrogen) and heated for 10 min at 90 °C. Electrophoresis and western blotting were performed as previously described^31^. The monoclonal antibodies used and their epitope on bank vole PrP were as follow: 9A2 (99–101), 12B2 (89–93), SAF84 (163–169), SAF32 (octarepeat).

## Additional Information

**How to cite this article**: Pirisinu, L. *et al.* Gerstmann-Sträussler-Scheinker disease subtypes efficiently transmit in bank voles as genuine prion diseases. *Sci. Rep.* **6**, 20443; doi: 10.1038/srep20443 (2016).

## Supplementary Material

Supplementary Information

## Figures and Tables

**Table 1:** Transmission of GSS cases in bank voles.

Inoculum *	Codon 129	PrP^res^ type (kDa)	Mean survival time ± SD (days)	Attack rate, n/N°^†^
#1-A117V	VV	6–7	95 ± 15	13/13
#2-A117V	MV	6–7	95 ± 8	11/11
Bv- #1-A117V°			107 ± 6	12/12
Bv- #2-A117V°			111 ± 5	11/11
#3-F198S	VV	8	143 ± 26	14/15
#4-F198S	MV	8	153	1/13
#5-P102L	MV	21 + 8	363 ± 38	6/8
#6-P102L-FC	MM	21 + 8	265‡; 705 ± 58§	8/12
#6-P102L-Cb	MM	8	153 ± 9	5/12
#7-P102L-FC	MV	8	208 ± 58	14/16
#7-P102L-Cb	MV	8	171 ± 53	13/13

^*^Human inocula were prepared from the frontal cortex. For two GSS P102L cases inocula were prepared from both the frontal cortex (FC) and cerebellum (Cb).

^†^n, Number of mice that developed confirmed TSE disease; N° number of inoculated mice.

^°^Second passages.

^‡^Survival time of a single Bv109I that accumulated 7 kDa PrP^res^.

^§^Survival time (mean ± SD) of Bv109I that accumulated 21 kDa PrP^res^.
