# Supplementary material for: Gerstmann-Sträussler-Scheinker disease subtypes efficiently transmit in bank voles as genuine prion diseases
Source: Sci Rep. 2016 Feb 4;6:20443. doi: 10.1038/srep20443 (PMC4740801; doi:10.1038/srep20443)
Supplement: Supplementary Information [file srep20443-s1.pdf]

Supplementary information

Gerstmann-Sträussler-Scheinker disease subtypes efficiently transmit in bank voles as genuine prion diseases

Laura Pirisinu[1], Michele A. Di Bari[1], Claudia D'Agostino[1], Stefano Marcon[1], Geraldina Riccardi[1], Anna Poleggi[2], Mark L. Cohen[3], Brian S. Appleby[3], Pierluigi Gambetti[3], Bernardino Ghetti[4], Umberto Agrimi[1], Romolo Nonno[1*]

[1]Department of Veterinary Public Health and Food Safety, Istituto Superiore di Sanità, Viale Regina Elena 299, 00161, Rome, Italy

[2]Department of Cell Biology and Neurosciences, Istituto Superiore di Sanità, Viale Regina Elena 299, 00161, Rome, Italy

[3]Department of Pathology, National Prion Disease Pathology Surveillance Center, Case Western Reserve University, School of Medicine, 2085 Adelbert Road Cleveland, Ohio, OH 44106, USA

[4]Department of Pathology and Laboratory Medicine, Indiana University School of Medicine, Indianapolis, IN 46202, USA

[*]**Corresponding author:**

Romolo Nonno, Department of Veterinary Public Health and Food Safety, Istituto Superiore di Sanità, Viale Regina Elena 299 00161, Rome, Italy

Telephone number: + 39 06 4990 2854

Email: romolo.nonno@iss.it

Table S1

**Patient ID #**	**PrP mutation**	**Codon 129**	**Sex**	**Age at onset (years)**	**Duration (years)**	**Clinical signs at onset**	**Neuropathology**
#1	A117V-129V	VV	M	29	~4	Ataxia, dysarthria	Multicore plaques in cerebral cortex; no SD[b]
#2	A117V-129V	MV	M	29	~5	Dysarthria, ataxia, mild cognitive impairment	Multicore plaques; no SD[b]
#3[a]	F198S-129V	VV	M	51	~6	Ataxia, jerky movements, dysarthria	Multicore plaques; NFT[c]
#4[a]	F198S-129V	MV	M	49	~10	Ataxia, dysarthria	Multicore plaques, fine SD[b], NFT[c]
#5	P102L-129M	MV	F	53	~1	Behavioral abnormalities, forgetfulness	Multicore and kuru-like plaques in the cerebellar and cerebral cortex, severe SD[b],
#6	P102L-129M	MM	M	53	~4	Motor and speech impairment	Multicore plaques, fine SD[b], NFT[c]
#7	P102L-129M	MV	M	41	~3.5	Impaired executive functions, emotional lability	Minimal abnormalities; rare small plaques; no SD[b]

[a] these two cases were from two brothers

[b] Spongiform degeneration

[c] Neurofibrillary tangles

Figure S1

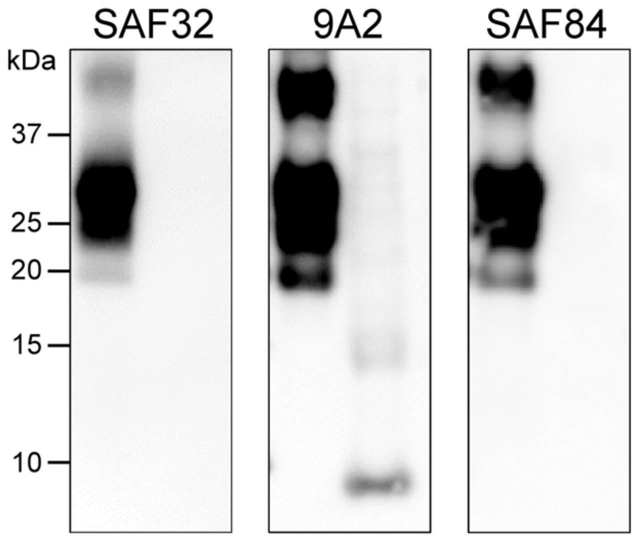

Epitope mapping of PrP^{res} from voles infected with GSS A117V.

Replica blots of PrP^{res} from a representative Bv109I infected with GSS A117V (lane 2 in each blot) were probed with different mAbs, as indicated on the top of each blot. The mAbs used recognize PrP epitopes in the N-terminal octarepeat region (SAF32), in the central domain (9A2, amino acid residues 99-101) and in the C-terminal globular domain (SAF84, amino acid residues 163-169). PrP^{res} from voles infected with GSS A117V was positive only with mAb 9A2, implying that the protease-resistant core of PrP^{Sc} consists of internal PrP fragments. PrP^{res} from a vole infected with classical scrapie, positive with all mAbs used, was loaded in the first lane of each blot as a control. The position of MW markers is indicated on the left of the first blot.

Figure S2

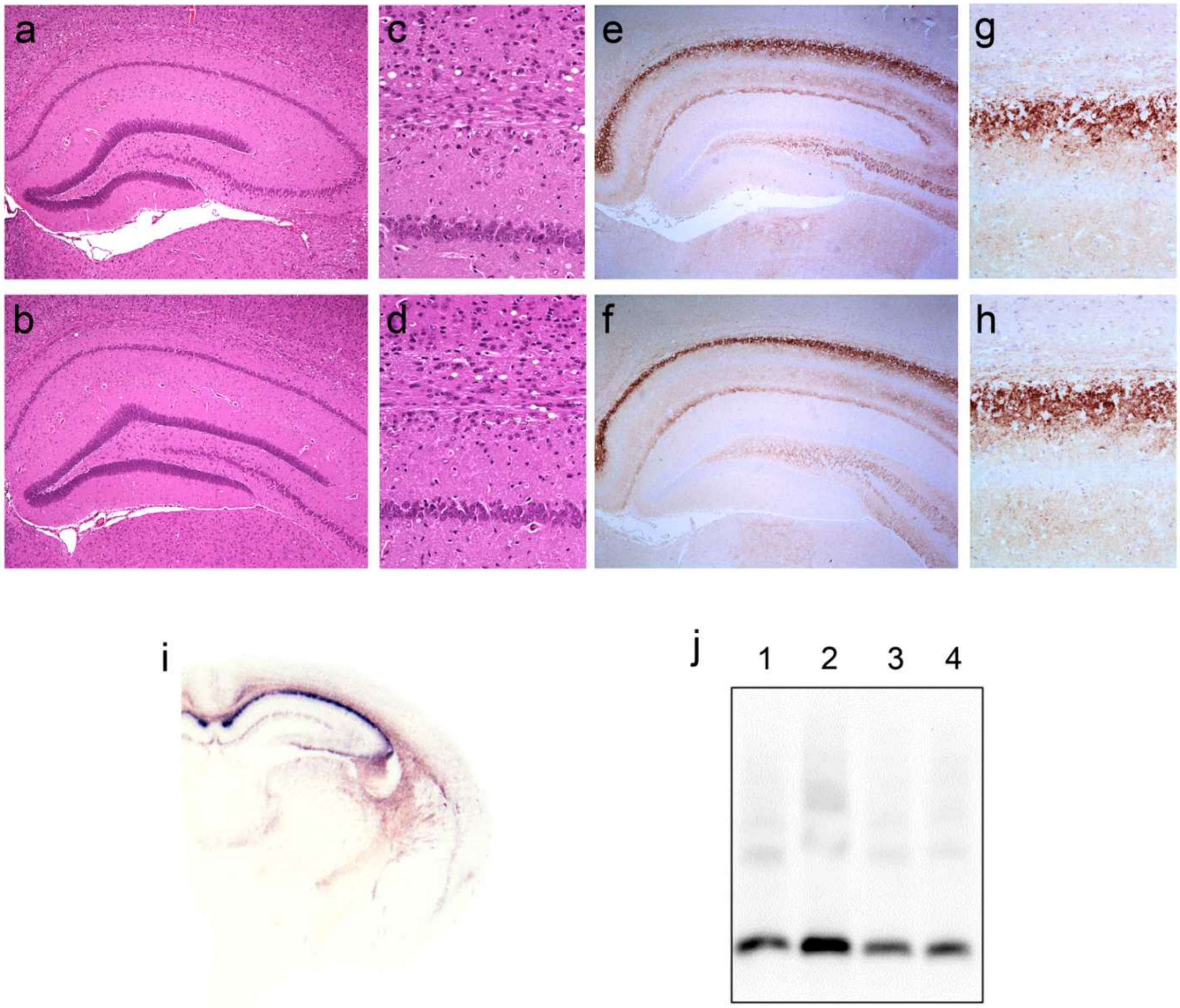

Neuropathology and PrP^{res} after second passage of GSS A117V cases in Bv109I.

Hippocampal sections from representative Bv109I after second passage of GSS A117V case #1 (**a, c, e, g**) and case #2 (**b, d, f, h**) were stained with haematoxilin and eosin (a, b, c, d) or analysed by IHC for PrP with mAb 6C2 (e, f, g, h). The neuropathological patterns observed were indistinguishable from those observed after primary passage (see Fig. 1). **i**, PET blot from a representative Bv109I after second passage of GSS A117V case #1. **l**, comparison of PrP^{res} from Bv109I after primary transmission (lane 1) or second passage (lanes 2-4) of GSS A117V case #1. The blot was probed with mAb 9A2.

Figure S3

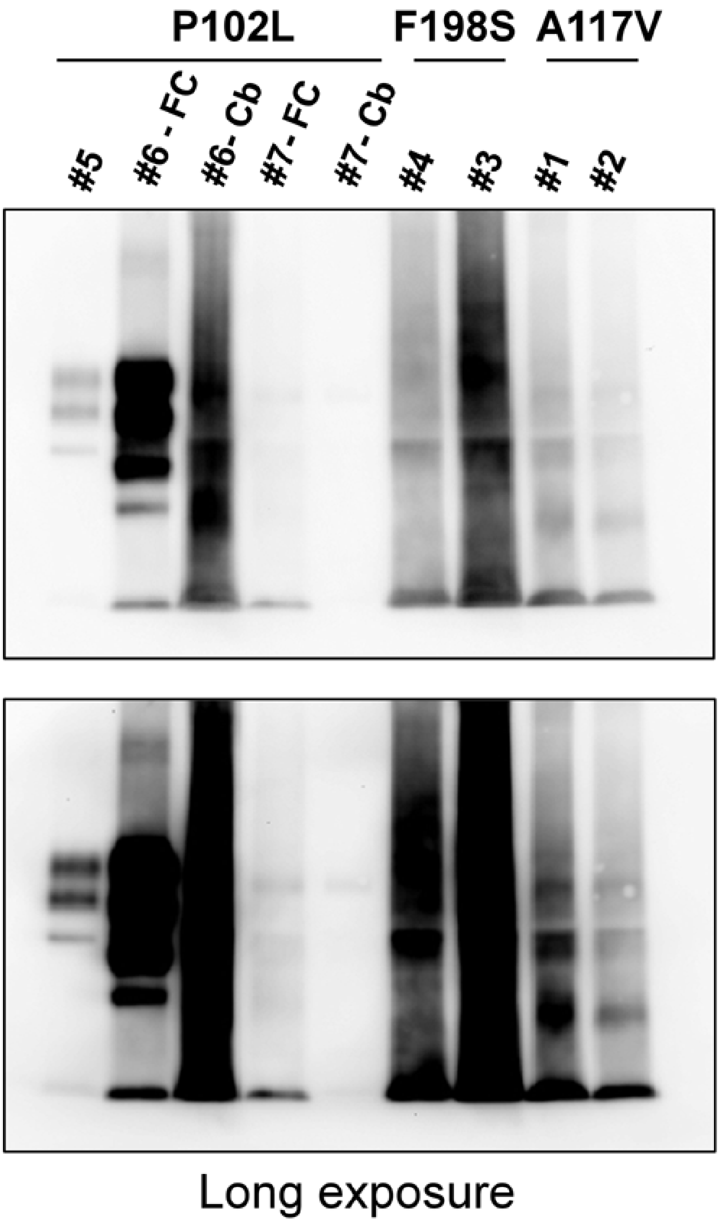

Comparison of PrP^{res} in the GSS inocula.

The inocula prepared from all GSS cases (indicated on the top of each lane according to Table 1) were compared for their PrP^{res} content. The inocula (10% brain homogenates in PBS) were added with an equal volume of 100 mM Tris-HCl containing 4% sarkosyl, treated with 100 µg/ml PK, added with an equal volume of isopropanol/butanol (1:1 v/v) and centrifuged at 20000 g for 5 min. The supernatants were discarded and the pellets were dissolved in denaturing sample buffer for WB analysis. All samples were loaded as 1,5 mg brain equivalent per lane. The blot was probed with mAb 9A2.

Figure S4

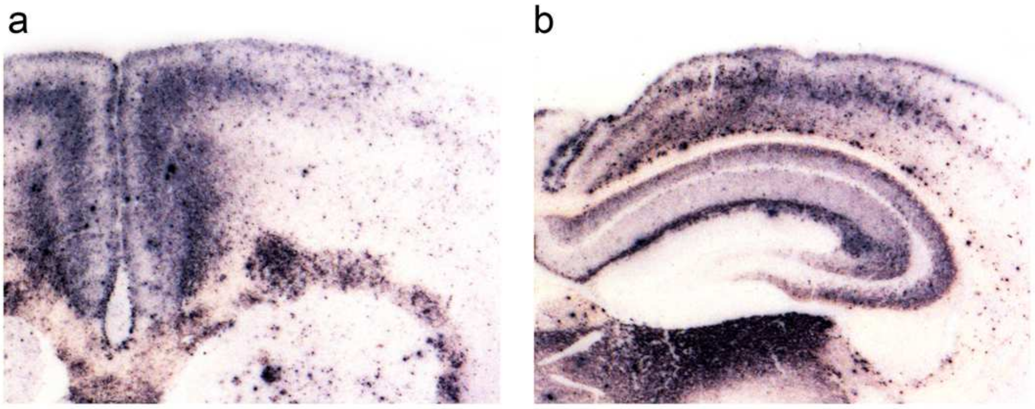

PET blot analysis of PrP^{Sc} from a Bv109I inoculated with frontal cortex from GSS P102L case #6 which accumulated 21 kDa PrP^{res}. Cortical (**a**) and hippocampal (**b**) sections show either diffuse PrP^{Sc} deposition and PrP^{Sc} plaques, whose distribution is similar to that observed by IHC in the same brain areas (see Fig. 3). The blot was probed with mAb 6C2.
